# Physical Methods for Seed Invigoration: Advantages and Challenges in Seed Technology

**DOI:** 10.3389/fpls.2016.00646

**Published:** 2016-05-12

**Authors:** Susana de Sousa Araújo, Stefania Paparella, Daniele Dondi, Antonio Bentivoglio, Daniela Carbonera, Alma Balestrazzi

**Affiliations:** ^1^Plant Cell Technology Laboratory, Instituto de Tecnologia Química e Biológica António Xavier, Universidade Nova de LisboaOeiras, Portugal; ^2^Department of Biology and Biotechnology ‘L. Spallanzani’, Universita degli Studi di PaviaPavia, Italy; ^3^Department of Chemistry, Universita degli Studi di PaviaPavia, Italy

**Keywords:** hormesis, ionizing radiation, magnetic field, microwaves, seed germination, seed vigor, ultraviolet radiation

## Abstract

In the context of seed technology, the use of physical methods for increasing plant production offers advantages over conventional treatments based on chemical substances. The effects of physical invigoration treatments in seeds can be now addressed at multiple levels, ranging from morpho-structural aspects to changes in gene expression and protein or metabolite accumulation. Among the physical methods available, “magneto-priming” and irradiation with microwaves (MWs) or ionizing radiations (IRs) are the most promising pre-sowing seed treatments. “Magneto-priming” is based on the application of magnetic fields and described as an eco-friendly, cheap, non-invasive technique with proved beneficial effects on seed germination, vigor and crop yield. IRs, as γ-rays and X-rays, have been widely regarded as a powerful tool in agricultural sciences and food technology. Gamma-rays delivered at low dose have showed to enhance germination percentage and seedling establishment, acting as an actual ‘priming’ treatment. Different biological effects have been observed in seeds subjected to MWs and X-rays but knowledge about their impact as seed invigoration agent or stimulatory effects on germination need to be further extended. Ultraviolet (UV) radiations, namely UV-A and UV-C have shown to stimulate positive impacts on seed health, germination, and seedling vigor. For all mentioned physical treatments, extensive fundamental and applied research is still needed to define the optimal dose, exposition time, genotype- and environment-dependent irradiation conditions. Electron paramagnetic resonance has an enormous potential in seed technology not fully explored to monitor seed invigoration treatments and/or identifying the best suitable irradiation dose or time-point to stop the treatment. The present manuscript describes the use of physical methods for seed invigoration, while providing a critical discussion on the constraints and advantages. The future perspectives related to the use of these approaches to address the need of seed technologists, producers and trade markers will be also highlighted.

## Introduction

One of the biggest challenges that humanity is now facing is improving the sustainability of agriculture while reducing its environmental impact, to meet the food demands of the growing global population (Edmondson et al., 2014). The idea of agricultural sustainability relies on the need to develop technologies and practices with no adverse effects on environmental goods and services but still leading to improvements in food productivity (Pretty et al., 2006). High vigor seeds are proxy of crop establishment and sustainable productivity. In 2012, the value of the EU seed market reached around € 7 billion corresponding to 20% of the global market ranking third after USA and China (Ragonnaud, 2013). In this economically competitive context, innovative biotech/molecular tools, treatments, or products are crucial to speed-up the consolidation process of the seed industry.

Despite contemporary agriculture largely uses chemical compounds, the use of physical factors might represent a good alternative to raise the yield of agricultural production while improving plant protection and storage (Aladjadjiyan, 2012). Physical methods for seed invigoration offer several advantages over conventional treatments based on chemical substances. First, they reduce the use of fertilizers, thus decreasing pollution of on-farm produced raw materials. Another advantage is that physical methods may be also used for seed disinfection before sowing and during the storage (Aladjadjiyan, 2012). Possible approaches include the treatment with electromagnetic waves (EWs), magnetic fields (MFs), the ultrasounds (US), and ionizing radiations (IR). Food treatment with IR, which enhances its microbiological safety and storability, has been one of the most studied technologies of the 20th century as a result of the general concern about the potential role of food irradiation in the safety of food supply (Farkas and Mohácsi-Farkas, 2011). Also, some of recent advances on the impact of IR on living organisms have been driven by the Space research. Among space factors, the ionizing radiation (including both gamma and X radiation) is one of the main growth constraints of organisms (Arena et al., 2014; Wolff et al., 2014). In this context, the cultivation of higher plants in space involves the development of new agro-technologies for the design of ecologically space greenhouses and a deep understanding of the effects of space factors on biological systems (De Micco et al., 2014). Importantly, the long term effects of the extraterrestrial environment on plant growth and development still needs to be fully addressed (Wolff et al., 2014).

The impacts of physical treatments in plants are being addressed at multiple levels, from morpho-structural aspects to changes in gene expression. Plant systems are excellent models for the characterization of biological responses to several environmental factors, which includes the genotoxic effects of physical and chemical reagents (Zaka et al., 2002; Balestrazzi et al., 2011; Confalonieri et al., 2014; Faè et al., 2014; Macovei et al., 2014). Physical treatments impacts in plants depend on several factors: radiation or MF (e.g., type, total dose, and dose rate) and plant features, such as species, cultivar, age, ploidy, and complexity of the target organ or tissue (De Micco et al., 2014). Interestingly, cumulative reports about the stimulatory effects of low dosages of a toxic agent, a phenomenon also known as hormesis, are emerging in many toxicological sciences (Luckey, 2006; Belz and Piepho, 2012; De Micco et al., 2014). Hormesis induced by physical or chemical stress factors has been described in all groups of organisms, including plants (Calabrese and Baldwin, 2000; Belz and Piepho, 2012). Seed irradiation with low doses of gamma (γ) rays has been reported to stimulate germination, plant growth, and synthesis of photosynthetic pigments (Kovács and Keresztes, 2002; De Micco et al., 2014; Macovei et al., 2014). Nevertheless, the same reports highlighted the damaging impacts of high radiation levels or prolonged exposure in living organisms.

‘Priming’ is a well-established treatment for enhancing seed quality throughout the transient activation of the pre-germinative metabolism that includes antioxidant functions and DNA repair processes (Paparella et al., 2015). Seed priming has emerged as an effective approach for increasing seed vigor and germination synchronization, as well as, seedling growth and field establishment under adverse environmental conditions (Ventura et al., 2012; Hussain et al., 2015). Low-vigor seeds can be improved using a wide range of treatments, some of them well characterized such as hydropriming and osmopriming. In the context of seed technology, physical methods have showed several advantages over conventional osmopriming protocols (Bilalis et al., 2012). One of the most investigated physical pre-sowing seed treatments in agriculture is based on the use of MFs (Aguilar et al., 2009). MFs have been described as eco-friendly, cheap, and non-invasive technique (Bilalis et al., 2012; Efthimiadou et al., 2014). Additionally, the impact of other types of physical treatments, such as, gamma (γ), and X ionizing, UV, and microwave (MW) radiation will be addressed in this review. Despite thermopriming being another important physical treatment used for improving seed vigor, it will not be covered here since it has been recently addressed by Paparella et al. (2015).

The present manuscript attempts for reviewing the use of physical methods for seed invigoration, while providing a critical discussion on the constraints and advantages. The future perspectives related to the use of these approaches to address the need of seed producers, trade markers and consumers will be also highlighted.

## “Magneto-Priming”: A Suitable Invigoration Protocol

The effects of MFs in living organisms have received considerable attention. Indeed, the Earth’s MF (50 μT) is a natural component of the environment (Belyavskaya, 2004). One Tesla (T) unit of magnetic flux density corresponds to 1 kg s^-2^A^-1^, where A stands for Ampere, the unit of electrical current or the current that flows with an electric charge of one Coulomb per second. Krylov and Tarakanova (1960) reported the effects of MFs in plants for the first time. In their work, a definition for magnetotropism came out when referring to the auxin-like effect exerted by MFs on germinating seeds. Recently, several reviews summarized the impacts of MFs on many biological processes in plants, such as growth, development, and metabolism (Maffei, 2014; Wolff et al., 2014). Both static magnetic field (SMF) and electromagnetic field (EMF) are used in agriculture for seed priming (the so called ‘magneto-priming’) with proven beneficial effects on seed germination, vigor and crop yield (Baby et al., 2011). Nevertheless, it should be pointed that SMFs differ considerably from EMFs. While SMFs are produced by a permanent magnet as in the case of the Earth’s magnetic field or by industrial processes, EMFs are generated by electrically charged objects being extended indefinitely throughout space (Mitchell and Cambrosio, 1997).

In the seed research context, the impacts of MFs on seeds have been studied with the overall goal to understand their suitability to derive new seed treatments. The beneficial effect of pre-sowing magnetic treatments for improving germination parameters and biomass accumulation has been described for a wide range of plants (**Table 1**) and recently reviewed by Teixeira da Silva and Dobránszki (2015). In these studies, different MF strengths have been tested ranging from 0 to 300 mT. Magneto-primed seeds showed improved germination rates, vigor and seedling biomass or root development. Another interesting feature of MFs-treatments is that they appeared to enhance tolerance to biotic (De Souza et al., 2006) or abiotic stresses (Javed et al., 2011; Anand et al., 2012) as result of the antioxidant response activation. Increased antioxidant enzyme activities of superoxide dismutase (SOD), catalase (CAT), and glutathione reductase (GR) were described in magneto-primed cucumber (*Cucumis sativus* L.) seeds (Bhardwaj et al., 2012). In agreement with these findings, Baby et al. (2011) highlighted a reduced production of superoxide radicals (O_2_^-^) in magneto-primed soybean (*Glycine max* (L) Merr. var Js-335) seeds. Consequently, MFs-treatments have the additional potentiality to be used for minimizing the drought- or disease-induced adverse effects on crop productivity.

**Table 1:** Summary of magnetic fields (MFs) effects on seed and seedling performance.

Species	Magnetic fields (MFs) applied	Effects described	Reference
*Triticum aestivum*	30 mT	No stimulation of seed germination, neither seedling growth. Increased antioxidant potential under soil flooding.	Balakhnina et al., 2015
*Tagetes patula*	100 mT	Improved germination, seedling vigor, and starch metabolism.	Afzal et al., 2012
*Glycine max*	200 and 150 mT	Increased germination parameters and seedling biomass. Plants with higher efficiency of light harvesting and biomass accumulation.	Baby et al., 2011; Shine et al., 2011
*Helianthus annuus*	50 and 200 mT	Increased germination and germination rate. Increased seedling length and biomass accumulation.	Vashisth and Nagarajan, 2010
*Vigna radiate*	5 mT	Improved germination, seedling vigor, and starch metabolism.	Reddy et al., 2012
*Solanum lycopersicum*	100 and 170 mT	Improved biomass and growth. Increased tolerance to biotic stresses.	De Souza et al., 2006
*Cucumis sativus*	200 mT	Improved germination, seedling vigor, starch, and anti-oxidative metabolism.	Bhardwaj et al., 2012
*Zea mays*	100 and 200 mT	Improved seedling growth, leaf water status, and photosynthesis in seedlings under soil water stress.	Anand et al., 2012
*Vicia faba*	0.1 mT	Improved seedling growth.	Rajendra et al., 2005
*Quercus suber*	0.015 mT	Improved sprouting rate and seedling biomass.	Celestino et al., 2000

Based on the previous assumption, some interest has been devoted to understand the physiological, biochemical, and molecular mechanisms underlying the improved performance of plants grown from MFs-treated seeds (Vashisth and Nagarajan, 2010; Baby et al., 2011; Javed et al., 2011; Anand et al., 2012). Vashisth and Nagarajan (2010) showed that, in sunflower (*Helianthus annuus* L.) seeds, exposure to magnetic fields (SMFs) appear to act like priming, with similar enhancement effects on seed performance. Indeed, these authors detected enhanced alpha-amylase, dehydrogenase and protease activities in magneto-primed seeds during imbibition when compared to non-treated ones. The higher activities of hydrolyzing enzymes were then linked to the enhanced seed germination, seedling vigor, and rooting traits of SMFs-treated seeds.

The mechanism by which plants perceive MFs and regulate the signal transduction pathway is not fully understood. Ahmad et al. (2007) suggested that MF-perception/signaling in plants is mediated by the blue light photoreceptors – cryptochromes. However, this aspect of magnetobiology still deserves in-depth investigation, as well as, the potential genotoxic side effects of MFs (Ghodbane et al., 2013). All these works highlighted the need for more studies to extend our knowledge on the molecular mechanisms involved in fastening seed germination, improving seedling vigor and enhancing photosynthetic capacity of magneto-primed plants.

Environmental factors, also known as interdependent factors, such as light, humidity and temperature are implicated in modulating horticultural and crop seed performance but the impact of their combined application with MFs remains unclear. This resulting knowledge could be relevant to develop new locally adapted seed treatments. Poinapen et al. (2013) studied how the combined impact of MFs and interdependent factors affected seed viability and performance in tomato (*Solanum lycopersicum* L. var. MST/32) under laboratory conditions. Relative humidity showed to be key factor modulating seed performance in magneto-primed seeds, especially during early stages of seed germination/imbibition. These findings are relevant for determining the most suitable combination of physical parameters to develop improved magneto-priming protocols.

## Ionizing Radiation Treatments

### Gamma Radiation Is a Promising Invigoration Approach

Ionizing radiation is a powerful tool in the field of agricultural sciences and food technology, being frequently utilized to address food microbiological safety and storability issues (Jayawardena and Peiris, 1988). Gamma (γ) radiation is a high-energy type of the IR able to penetrate and interact with living tissues. It is usually delivered by means of Cobalt-60 (^60^C) sources (Moussa, 2006). The absorbed dose of IRs is expressed in units of Gray (Gy), in which 1 Gy dose corresponds to 1 Joule radiation energy adsorbed per kilogram. When interacting with biological material (organism, organ, tissue), the absorbed dose of IRs could be also expressed in Sievert units (Sv), in which 1 Sv dose corresponds to 1 Joule radiation energy adsorbed per kilogram of organ or tissue weight. Another key parameter for establishing IR treatments is the dose rate (rate of energy deposition, expressed as Gy h^-1^). Currently, γ-rays are predominantly used in situation in which a high sterilization level is required. Gamma-irradiation treatments are widely used to abolish microbial contamination or control insect pests and pathogens, thus acting in disease prevention. Besides safety issues, γ-irradiation is also used to delay fruit ripening and vegetable sprouting by hampering the activation of key enzyme activities, contributing to extended crop shelf-life (Mokobia and Anomohanran, 2005; Moussa, 2006). In another agricultural context, γ-rays represent an effective mutagenic tool for plant breeders who want to add new traits into commercially valuable crops and develop new varieties (Irfaq and Nawab, 2001).

Among the different radiobiology aspects, the characterization of the γ-rays effects on seeds is a topic that is recently receiving evident attention. Studies undertaken have been particularly focused on the impacts of low dose rate and/or low total dose γ- irradiation on germination yield and seedling performance. Gamma-rays directly interact with the cell components at multiple levels, reaching membranes, proteins, and nucleic acids (Kovács and Keresztes, 2002). However, an indirect action is also well reported through the generation of reactive oxygen species (ROS) from water radiolysis (Borzouei et al., 2010; Esnault et al., 2010). ROS diffuse and damage cellular macromolecules and organelles. Nevertheless, the biological effects γ-rays have been demonstrated to be strongly dependent on the intensity, dose-rate and exposure time. Concerning seed treatments, γ-rays delivered at low dose enhance germination percentage and seedling establishment, acting like an actual ‘priming’ treatment.

In the context of seed technology, the positive impact of seed γ-irradiation as seed invigoration treatment has been the driving force for investigating the molecular mechanism activated in seeds response to this physical treatment. Recently, Qi et al. (2015) investigated the impact of γ-rays treatments on *Arabidopsis thaliana* seeds by assessing the biological responses of resulting seedlings. Seed irradiation with total doses lower than 100 Gy notably stimulated germination index. The same feature was observed concerning seedling growth, primary root length and fresh weight compared with the non-irradiated seed lots. Among tested irradiation doses, *A. thaliana* seeds treated with the 50 Gy dose displayed the maximal positive effects on all the tested growth parameters. Gamma-irradiation proved also to be an efficient approach to improve seed germination performance and seedling establishment in crops or woody species. Maity et al. (2005) described γ-ray-induced effects on *Oryza sativa* L. cv-2233 and *Phaseolus mungo* L. seeds. Radiation applied ranged the 50–350 Gy dose. While irradiation at lower doses improved morphological traits like plant height, shoot number, panicle length and seed number per panicle, the exposure at higher doses had a negative impact on the same parameters. In *O. sativa*, the most stimulatory dose corresponded to 50 Gy but for *P. mungo* the most enhanced beneficial effect was noticed only at 200 Gy. The impact of gamma irradiation was also studied on maize (*Zea mays*, hybrid Turda Star) seeds (Marcu et al., 2013). In this study, a radiation sensitivity test was performed for comparing germination capacity, plant growth and content of photosynthetic pigments between irradiated and non-irradiated seeds. Again, the stimulatory effects of γ-ray were seen at low doses (2–30 Gy) while high doses (70 Gy) showed to be harmful to plant performance. One important outcome of this study is that low dose irradiation has also a beneficial effect on the productive traits in crops, besides the germination or seedling establishment. In okra [*Abelmoschus esculentus* (L.) Monech], Hegazi and Hamideldin (2010) compared the effects of two pre-sowing seed treatments: conventional hydropriming and γ-irradiation performed at different doses (300, 400, and 500 Gy). In hydropriming treatments, seed are just soaked in water before radicle emergence takes place, allowing by this way the activation of the pre-germinative metabolism. Although both pre-sowing treatments were effective in improving plant establishment, γ-irradiation with 400 Gy provided the best results in terms of germination rate, seed yield and quality, as well as, photosynthetic capacity. Altogether these results highlight that the application of physical seed treatments is dependent of the species studied, requiring a previous assessment of the optimal doses to be used.

The beneficial effects of γ-rays treatments were not restricted to crops species, constituting actually an alternative approach to support native species conservation. The effects of gamma irradiation on *Moluccella laevis* L. (bells of Ireland) seeds were investigated (Minisi et al., 2013). Similarly to previous descriptions, γ-rays delivered at low doses (up to 5 Gy) increased germination percentage when compared with non-irradiated or irradiated samples with higher doses (up to 20 Gy). Low dose-γ-rays have also stimulated seed germination, vigor and seedling growth in wild oat (*Avena fatua* L.) (Maherchandani, 1975), garden cress (*Lepidium sativum* L.) (Majeed et al., 2010), deadly nightshade (*Atropa belladonna* L.) (Abdel-Hady et al., 2008), okra (*Abelmoschus esculentus* L. Monch.) (Dubey et al., 2007), and rocket *(Eruca vesicaria* L. subsp. sativa) (Moussa, 2006). All these works provided cumulative evidences those small doses of γ-rays results in a beneficial action in physically treated seeds, which fits the definition of hormesis (Luckey, 1980, 2006). The low dose threshold is usually placed just above the natural radiation background levels (2.4 milliSievert per year, in which 1 Sievert represents the equivalent biological effect of the deposit of a joule of radiation energy in a kilogram of human tissue) and defined by the frontier between biopositive and bionegative effects. Hormesis is a fundamental concept in evolutionary biology (Calabrese and Baldwin, 2000). It represents an adaptive response of cells and organisms to moderate and intermittent stresses, stimulating the activation of cellular defense and repair mechanisms against the stressing agent, otherwise missing in the absence of stress (Mattson, 2008).

The biological and molecular mechanisms underlying the beneficial effects of radiation hormesis are still debated but several clues have already been proposed. Several authors pointed at ROS produced in the seed as key regulators of the response to γ-rays, since they act as signaling molecules, triggering and amplifying stress and antioxidant responses. Consequently, irradiated plants could easily overcome daily stress factors, such as fluctuations in the light intensity, temperature variations, and water loss during growth (Maherchandani, 1975; Fan et al., 2003; Gicquel et al., 2012; Qi et al., 2015). Sjodin (1962) and Abdel-Hady et al. (2008) claimed that low dose γ-irradiation exert a stimulating effect on enzymatic activity, as well as, nucleic acids and proteins synthesis in treated seeds. Based on this, it is not surprising that the consequence is a boost in the embryo/seed metabolism leading to early dormancy breaking, germination and plant development. In another study, the impact of acute and chronic gamma-irradiation on plant genome stability and global genome expression was analyzed (Kovalchuk et al., 2007). The transcriptomic data obtained showed that a differential response between acute or chronic treatments is observed. In response to acute radiation, plant metabolism is shifted to immediate repair of the damage, activation of pro-survival mechanisms and possibly the inhibition of cell division/cell differentiation. In contrast, chronic irradiation triggered a totally different response, in which the expression of genes belonging to general stress and nucleic acid metabolism categories is activated. Moreover, the outcomes of this study also showed that chromatin modifications and changes in methylation patterns are associated to an adaptation to chronic irradiation, both having a trans-generational nature.

All above mentioned studies provided cumulative evidences that gamma-irradiation is a valuable tool in seed technology, since low-dose treatment can act efficiently as a seed invigoration treatment. However, more studies are needed to understand the molecular basis underlying the improved growth/development response observed in irradiated seeds. Despite the advantageous aspects mentioned, gamma-irradiation treatments in a seed technology context depend on the proper establishment of γ-irradiation facilities able to work at an industrial scale. The application of this seed invigoration method still requires an extensive research in order to define the optimal treatment conditions (total dose, dose rate) which depend on crop species, genotype and environmental context.

### Effects of X-Rays in Seed Germination and Seedling Development

The effects of X-rays on organisms are still not fully understood. Among IR sources, X-rays have a wavelength in the range of 0.01 to 10 nm of the electromagnetic spectrum, corresponding to frequencies in the range 30 to 30000 PHz (1 Petahertz corresponds to 10^15^ Hertz) and energies in the range 120 eV to 120 keV (Kotwaliwale et al., 2014). Soft X-rays with energies of about 0.12 to 12 keV are the most suitable X-radiation to be used on agricultural products, due to low penetration power and ability to reveal the internal density changes (Kotwaliwale et al., 2014). Food treatment with X-rays has been performed to improve food microbiological safety and storability (Farkas and Mohácsi-Farkas, 2011). While the impact of X-rays is well studied in humans due to its general use in medical practice, research on the impact of X-radiation on plants had only a boost during the 1930–1960 timeframe (Benedict and Kersten, 1934; Bless, 1938; Smith, 1950; Caldecott et al., 1952; Yagyu and Morris, 1957; Beard et al., 1958).

To the best of our knowledge, very few works focused on the impacts of X-ray irradiation on seed performance were published after the 60′s decade (Al-Enezi et al., 2012; De Micco et al., 2014; Einset and Collins, 2015; Pérez-Torres et al., 2015). Increasing X-ray irradiation doses were seen to reduce seed germination percentage and root growth of date plam (*Phoenix dactylifera* L.) seeds (Al-Enezi et al., 2012). However, these authors noticed a stimulatory effect on leaf growth when seeds were irradiated with a 0.65 Gy dose. Another interesting study was recently conducted in *S. lycopersicum* L. cv. Microtom (De Micco et al., 2014). In this study, anatomical and ecophysiological features (e.g., growth traits, leaf anatomy) of tomato plants grown from seeds irradiated with increasing X-rays doses were investigated. The overall results showed that germination and development of functional leaves was not significantly hampered by increasing irradiation dose, which suggested some resistance of this cultivar to irradiation. Further, the radio-resistance of the Microtom cultivar was supported by the slight structural perturbations observed in leaves with minor impairment of the photosynthetic efficiency, namely when seeds were irradiated with high doses of X-rays. The identification of radio-resistant candidate species or seed lots can be an important achievement for the design of space-oriented agriculture (Arena et al., 2014).

The current knowledge about the effects of X-rays in plants is still scarce, focused on few aspects of the plant physiology and needs to be extended. New clues about molecular and physiological mechanisms underlying the resistance of plant tissues to X-rays could be unveil by using global profiling techniques (e.g., Omics).

## “Priming” Seeds with Ultraviolet Radiation: A Difficult Task

The amount of solar UV radiation reaching the Earth’s surface is increasing as consequence of the depletion of the stratosphere ozone layer. According to the wavelength range, solar UV radiation is classified into UV-C (200–280 nm), UV-B (280–320 nm), and UV-A (320–400 nm). A large number of studies have been focused on the global impact of UV exposure on plants at multiple levels, from the ecosystem to the whole plant (Kovács and Keresztes, 2002; Heisler et al., 2003). On the other hand, very few studies have been dedicated to investigate UV impact in seed biology and germination (see **Table 2**).

**Table 2:** Summary of ultraviolet (UV) effects on seed and seedling performance.

Species	UV applied	Effects described	Reference
*Lactuca sativa*	UV-C	Enhanced NaCl stress tolerance.	Ouhibi et al., 2014
*Phaseolus vulgaris*	UV-C	Increased germination rate. Increased accumulation concentration of bioactive molecules in seed coats.	Guajardo-Flores et al., 2014
*Vigna radiata*	UV-C; UV-A	Increased germination rate and seedling vigor. Seedlings with reduced susceptibility to root infecting fungi.	Hamid and Jawaid, 2011; Siddiqui et al., 2011
*Arachis hypogaea*	UV-C	Increased germination rate and seedling vigor. Seedlings with reduced susceptibility to root infecting fungi.	Siddiqui et al., 2011
*Vigna mungo*	UV-B	Exposure of hydrated seeds significantly reduced germination, suppressed the synthesis of photosynthetic pigments, as well as, root, and shoot development. Curling and twisting of the seedlings.	Shaukat et al., 2013
*Carthamus tinctorius*	UV-B	Enhanced germination rate but subsequent growth of the seeedlings is hampered.	Farokh et al., 2010

Although UV-C radiation is extremely harmful to organisms, it is not relevant under natural conditions of solar irradiation (Hollósy, 2002). UV-C radiation is non-ionizing and it penetrates superficially into the plant tissues, which supports its potential as a germicidal agent. Seed treatments with low doses of UV-C (3.6 kJ m^-2^) were used to elicit host resistance to black rot in cabbage (*Brassica oleracea* L.) (Brown et al., 2001). This UV-C seed treatment also improved the quality and growth response of cabbages under greenhouse conditions. In another study, Ouhibi et al. (2014) investigated the impact of UV-C pre-sowing treatments in lettuce (*Lactuca sativa* L. ‘Romaine’). Lettuce seeds were UV-C- treated by exposure to 0.82 and 3.42 kJ m^-2^ doses and resulting seedlings were challenged with salt stress. The results showed that UV-C treated seedlings were able to mitigate the impact of excessive salinity, possibly as result of the enhanced free radical scavenging activity detected in their leaf tissues (Ouhibi et al., 2014). Additionally, the authors also showed that a dose-dependent response occurs: seedlings derived from seeds treated with the lowest UV-C dose showed higher tolerance to salinity conditions.

Despite UV-B radiation represents only approximately 1.5% of the total spectrum, the UV-B harmful effects on plant physiology are well described. Among them, DNA damage, proteins and membranes injury which limits photosynthesis and plant growth (Hideg et al., 2013; Choudhary and Agrawal, 2014). The effects of UV-B irradiation on seed germination, seedling growth and plant development were investigated in mash-bean (*Vigna mungo* (L.) Hepper) (Shaukat et al., 2013). Although the authors reported an accelerated germination rate, the final germination percentage remained unaffected by the UV-B treatment. Importantly, some deleterious effects were evident, such as the reduction in root and shoot growth. At the biochemical level, the UV-B treatment triggered a significant increase in total soluble phenols, as well as, an enhancement of the activities of L-phenylalanine ammonia lyase and tyrosine ammonia lyase. The lack of information available about the possible use of UV-B radiation as a seed invigoration treatment may reflect its unsuitability for the purpose.

Ultraviolet-A radiation represents approximately 6.3% of the incoming solar radiation and it is the less hazardous component of UV radiation (Hollósy, 2002). The information about the possible use of UV-A radiation as seed invigoration treatment is very scarce. Hamid and Jawaid (2011) investigated the effects of UV-A radiation on mung bean (*Vigna radiata* L.) seeds. The results of this study were very promising, showing that pre-sowing UV-A treatment stimulated germination rate, as well as, seedling performance reflected in the values of specific leaf area, root and shoot length and dry weight. To the best of our knowledge, this is the only study publicly available but this positive result supports further research developments on this topic.

The impacts of increasing solar UV radiation on terrestrial ecosystems have been extensively reviewed under the present Climate Changes context. Similarly to what is described for MFs, the impact of UV irradiation can significantly differ, depending on the target organism and radiation component used. Positive effects of UV- A and UV-C radiation have been highlighted on seed germination/seedling vigor, as well as, seed health. However, a deeper insight is still requested to elucidate about the molecular mechanisms underlying the improvements occurring in the UV-treated seeds.

## Microwaves Potentialities in Seed Technology

Microwaves (MWs) are components of the electromagnetic spectrum. MWs include radiation ranging in frequency from 300 MHz (300 million cycles per second) to 300 GHz (300 billion cycles per second), which correspond to a wavelength range from 1 m down to 1mm (Banik et al., 2003). Despite the initial controversy, it is now generally accepted that the absorbed non-ionizing electromagnetic radiation as MWs induces thermal and non-thermal effects in biological systems (Banik et al., 2003). Mounting evidences show that MWs cause different biological effects depending on field strength, frequencies, wave forms, modulation and duration of exposures (Vian et al., 2006). While the effects of MWs on humans and animals were widely investigated, a very limited number of published studies have addressed the MWs-mediated effects on plants (Jayasanka and Asaeda, 2014). Interestingly, most of the studies currently available describe the impact of the 2.45 GHz radiation, which is absorbed by water molecules in living cells (Iuliana et al., 2013). When the MW radiation is absorbed by living tissues, it causes ionic movement, dipole rotation and distorsion of the electron orbit which ultimately results into fast and selective heating.

In a seed technology context, non-lethal MWs treatments have been extensively used for seed disinfection before sowing or storage (Reddy et al., 1995, 1998; Scialabba and Tamburello, 2002; Aladjadjiyan, 2010; Knox et al., 2013). Interestingly, deleterious MWs treatments have been used for inhibiting germination of weed seeds buried in the soil (Velázquez-Martí et al., 2006; Sahin, 2014). MWs treatments applied caused soil heating (up to 80°C) and weed germination was totally inhibited. Consequently, MWs emerged as a valid non-chemical alternative for weed control in greenhouses from horticultural/ornamental plant nurseries.

The knowledge about the effects of MWs as seed invigoration treatment, as well as, germination performance is limited and restricted to few plants (**Table 3**). Soran et al. (2014) investigated impacts of MWs at bands corresponding to wireless router (WLAN, Wireless Local Area Network: 70 mW m^-2^) and mobile devices (GSM, Global System for Mobile communication: 100 mW m^-2^) in three aromatic species. Parsley (*Petroselinum crispum* L. cv. Plained Leaved 2), celery (*Apium graveolens* L. cv. Pascal Giant), and dill (*Anethum graveolens* L. subsp. *hortorum* cv. Common) seed were irradiated and used to assess the impacts on leaf anatomy, essential oil content, and volatile emissions. The results showed that the applied MWs treatments induced structural (e.g., thinner cell walls and smaller plastids) and chemical modifications (e.g., enhanced emission monoterpenes) on the three plant species studied. Interestingly, the WLAN-frequency MWs appeared to more harmful than GSM-frequency MWs on the above measured parameters, despite the higher radiation value.

**Table 3:** Summary of microwaves (MWs) effects on seed and seedling performance.

Species	MWs applied	Effects described	Reference
*Lens culinaris*	2.45 GHz	No impact on seed germination or rate. Seedling length is stimulated after exposure of 30 s. Higher exposure time affects negatively all parameters.	Aladjadjiyan, 2010
*Raphanus sativus*	10.5 and 12.5 GHz	Reduced germination, germination rate, and hypocotyl growth rate.	Scialabba and Tamburello, 2002
*Oriza sativa*	2.45 GHz	Increased the germination percentage and rate, as well as, the primary shoot, and root length.	Talei et al., 2013
*Triticum aestivum*	2.45 GHz	Reduction of seedborne *Fusarium graminearum* infestation, and seed vigor.	Reddy et al., 1998
*Solanum tuberosum*	2.45–54 GHz	Highest biomass growth in seed potato germs.	Jakubowski, 2010
*Hordeum vulgare*	2.45 GHz	Enhanced germination and vigor index after exposure during 20 s.	Iuliana et al., 2013

While the 2.45 GHz MWs radiation has no clear effect in seed germination, it seems to be beneficial on seedling growth and biomass accumulation in different species (Aladjadjiyan, 2010; Jakubowski, 2010; Talei et al., 2013). As described for other types of radiation treatments, the effects of MWs on seeds depend on the plant species and growth stage, as well as, exposure duration, frequency, power density (Jayasanka and Asaeda, 2014). The number of studies available is still not sufficient to evaluate the impact of MWs in plant systems, neither in seed technology.

The most promising approach for MWs treatments should rely on the design new chemical-free approaches to control weeds in agro-industrial facilities and disease control in seed storage and seed production systems.

## Physical Methods to Study the Impact of Physical Seed Invigoration: Electron Paramagnetic Resonance as a Case Study

Electron paramagnetic resonance (EPR), also known as electron spin resonance (ESR), is technique for the qualitative and quantitative analysis of short-lived (10^-9^–10^-1^ s) free radical species, such as ROS. This powerful tool, currently regarded as one of the most specific and sensitive for this purpose, rise from the original studies in quantum mechanics carried out by Zeeman (1897). Subsequently, these studies were interpreted by Uhlenbeck and Goudsmit (1926), who introduced the concept of ‘spin’ (quantitized angular moment) as intrinsic feature of the electron. Although the first EPR spectrum was reported by Zavoisky (1945), EPR was only made available to the research community in the 1980s when the equipment became cheaper and commercially available. This was essential to support research on biological systems containing organic-based radicals and transition metals (Sahu et al., 2013).

An EPR active system includes, at least, a single unpaired electron spin located within a molecular orbital. The electron can exist in two alternative quantum states (*M* = ±1/2). In the absence of MF, the two quantum states possess the same energy. When a MF is applied, the energy of the -1/2 state decreases and the energy of the +1/2 state increases, depending on the strength of the MF. Unpaired electrons can change their spin state and these events associated with energy absorption are monitored and converted into a spectrum (for EPR fundamentals, see Weil and Bolton, 2006). EPR resolution can be further enhanced using tracer molecules (spin probes), which are artificially introduced in the target biological systems. The tracer molecules are stable diamagnetic compounds able to bind the short-lived ROS and to originate long-lived radical species (also known as paramagnetic spin adducts). The latter accumulate in cells during minutes or hours, facilitating EPR-based detection (Sahu et al., 2013). EPR spectroscopy is currently used *in planta* to dissect and understand the multiple roles played by ROS in plant growth regulation and stress response (Steffen-Heins and Steffens, 2015).

The potential of this tool for the high-resolution profiling of radicals in seeds still needs to be fully exploited. From an historical perspective, EPR significantly contributed to understand the role of free radicals in seed deterioration, providing for the first time seed-specific radical species spectra. An EPR signal, corresponding to an unknown organic radical, was initially detected in aged soybean seeds (Priestley et al., 1985) and subsequently confirmed in desiccated maize seeds (Leprince et al., 1990, 1995). In these early studies, the influence of oxygen and temperature on ROS-mediated injury at the seed level was also revealed by EPR spectra. An intriguing aspect of seed viability explored by EPR is related to the changes in mobility or viscosity of molecules (e.g., soluble sugars) occurring when the cytoplasm enters a glassy state. This phenomenon occurs when seeds are stored under low water and/or low temperature conditions and it has been monitored using spin probes. Spin probes molecular mobility can be fast or slow depending on their distribution within the different seed components. Indeed, nitroxide spin probes were successfully used to establish a correlation between molecular mobility, occurrence of intracellular glasses and seed storage stability (Buitink et al., 1998, 1999; Leprince and Hoekstra, 1998). Moisture- and temperature-dependent changes occurring in pea (*Pisum sativum* L.) and *Impatiens* seeds are correlated with aging rate and this is reflected in the rotational motion of a spin probe located in the cytosol (Buitink et al., 2000).

Electron paramagnetic resonance was successfully used for the functional analysis of plasma membranes integrity in seeds (Smirnov et al., 1992). These authors used a nitroxide spin probe to investigate the stability of cellular membranes in wheat embryos. The results showed that the cell membrane was semi permeable to nitroxide molecules in seeds with ≥13% moisture content. When seeds were submitted to conditions of artificial aging, the membrane function/permeability was definitely compromised and this aspect was reflected in EPR spectra acquired. Another study conducted also conducted in wheat seeds by Golovina et al. (1997) also corroborated the effects of seed aging on embryo membrane integrity. Besides revealing that plasma membrane permeability of embryo axes increased more rapidly during aging than in other tissues, the study also correlated the lack of membrane integrity with loss of germination. The key role of membrane permeability integrity during imbibition in chilling-sensitive neem (*Azadirachta indica* L.) seeds was also investigated using a nitroxyl spin probe technique (Sacandè et al., 2001).

Paramagnetic species profiles are emerging as promising biomarkers for screening stress tolerance in cereal grains. Labanowska et al. (2012) used EPR to analyze the long lived stable radicals stabilized by starch and other carbohydrates present in grains of five wheat genotypes with different drought stress tolerance. Increased amounts of carbohydrate and semiquinone radicals were found in seeds from drought sensitive genotypes, which also correlated with higher amounts of starch determined by biochemical analysis. These authors also proposed that those EPR radical profiles can be used as indicators of stress tolerance. Kurdziel et al. (2015) also used EPR to investigate free radical profiles in wheat whole grains but also in grain specific components (embryo, seed coat, and endosperm) from four wheat genotypes with different degree of drought tolerance. Again, the level of carbohydrate radicals was significantly higher in the drought-sensitive genotypes, corroborating their use as a reliable marker of the plant ability to withstand water deficit. More recently, EPR was used by Labanowska et al. (2016) to investigate the influence of short-term ozone application on wheat, oat and barley grains in two contrasting genotypes in terms of oxidative stress tolerance. EPR revealed that the character and the number of paramagnetic species [transition metal ions: Fe(III), Cu(II), Mn(II), and stable organic radicals] changed upon ozone exposure, depending on the cereal species, stress tolerance of a particular genotype and the part of grain studied. Moreover, the patterns of stable organic radicals (semiquinone, phenoxyl, and carbohydrate) significantly changed in response to ozone treatment, showing a stronger enhancement of these paramagnetic species in the embryo of the tolerant cultivars. Interestingly, oxidized iron species (Fe^2+^) in the embryo were stabilized by the organic matrix while in seed coat and endosperm increased free radical levels correlated with the amount of transition metal ions (Fe^2+^, Cu^2+^, Mn^2+^). The results obtained from this study also corroborated the suitability of EPR to screen oxidative stress tolerant cultivars of cereals.

The knowledge so far acquired through EPR-based studies in food processing or seeds, as mentioned previously, support the application of this technique to investigate the effects of physical invigoration methods on seeds. ESR was used to investigate the biochemical changes induced in maize seeds upon irradiation with increasing doses of gamma-radiation (Marcu et al., 2013). In this work, a correlation between the relative concentrations of paramagnetic species as a function of the absorbed irradiation dose was established. This reflects the increase of ROS generated through water radiolysis. The results suggest that EPR spectrometry is a relatively fast and simple technique to measure free radicals generated upon irradiation. This has an enormous potential on a seed technology context, since EPR can be used to monitor seed invigoration treatments and identify the best suitable irradiation dose or time-point to stop the treatment.

## Conclusion

High vigor seeds are proxy of crop establishment and sustainable productivity. Physical seed invigoration methods are an alternative approach – to current chemical based – to develop new biotech-based solutions for the growing world seed market. The use of physical methods for increasing seed germination and seedling vigor offers eco-friendly advantages and the possibility to be used in a high throughput scale. Promising approaches include the treatment with MFs, MWs, and IRs.

Besides the existence of adequate facilities to perform the physical treatments, the current gap of knowledge on the pre-germinative metabolism is hampering its successful application of priming treatments, as seen for chemical treatments. So far, the research conducted to understand the molecular mechanisms governing physical seed invigoration lacks deepness. Most of the studies were restricted to the assessment of the impact of a range of radiation treatments applied, with very little information on biochemical or gene expression changes occurred. The lack of biomarkers associated with the best suitable irradiation dose or time-point to stop the treatment is hampering the implementation of physical seed invigoration protocols at the industry level.

Nevertheless, researchers from Academia and Industry are currently focused on overcoming these issues. The recent advances on molecular high-throughput techniques (e.g., Omics) combined with the recent release of new genomic resources on target species or crops are expected to lead, in a short-term, research on this topic. More studies are needed to indentify the molecular players triggered during the seed response to physical invigoration treatments, especially in radio-tolerant species or cultivars. On the other hand, by expanding the number of species/genotypes tested with each different approach, it will be possible to identify those targets best suitable for a specific physical treatment, preventing deleterious conditions. The study of the impact of the environmental conditions in modulating the response of seed to radiation treatments could not be neglected, constituting it self a promising research avenue. This knowledge is crucial to develop new strategies to design new biotech-based treatments to modulate and improve seed germination and invigoration.

An integrated and multi-disciplinary approach is needed to speed up basic and translational research in seed technology, finally producing guidelines for the seed operators.

## Author Contributions

SSA and AB conceived the topic. All authors wrote, revised, and agreed with contents of the final manuscript.

## Conflict of Interest Statement

The authors declare that the research was conducted in the absence of any commercial or financial relationships that could be construed as a potential conflict of interest.

## References

[B1] Abdel-Hady M. S. Okasha E. M. Soliman S. S. A. Talaat M. (2008). Effect of gamma radiation and gibberellic acid on germination and alkaloid production in *Atropa belladonna* L. *Aust. J. Basic Appl. Sci.* 2 401–405.

[B2] Afzal I. Mukhtar K. Qasim M. Basra S. M. A. Shahid M. Haq Z. (2012). Magnetic stimulation of marigold seed. *Int. Agrophys.* 26 335–339. 10.2478/v10247-012-0047-1

[B3] Aguilar C. H. Dominguez-Pacheco A. Carballo A. C. Cruz-Orea A. Ivanov R. Bonilla J. L. L. (2009). Alternating magnetic field irradiation effects. *Acta Agrophys.* 14 7–17.

[B4] Ahmad M. Galland P. Ritz T. Wiltschko R. Wiltschko W. (2007). Magnetic intensity affects cryptochrome-dependent responses in *Arabidopsis thaliana*. *Planta* 225 615–624. 10.1007/s00425-006-0383-0 16955271

[B5] Aladjadjiyan A. (2010). Effect of microwave irradiation on seeds of lentils (Lens Culinaris, Med.). *Rom. J. Biophys.* 20 213–221.

[B6] Aladjadjiyan A. (2012). “Physical factors for plant growth stimulation improve food quality,” in *Food Production - Approaches, Challenges and Tasks*, ed. Aladjadjiyan A. (Rijeka: InTech), 145–168.

[B7] Al-Enezi N. Al-Bahrany A. Al-Khayri J. (2012). Effect of X-irradiation on date palm seed germination and seedling growth. *Emirates J. Food Agric.* 24 415–424.

[B8] Anand A. Nagarajan S. Verma A. P. S. Joshi D. K. Pathak P. C. Bhardwaj J. (2012). Pre-treatment of seeds with static magnetic field ameliorates soil water stress in seedlings of maize (*Zea mays* L.). *Indian J. Biochem. Biophys.* 49 63–70. 22435146

[B9] Arena C. De Micco V. Macaeva E. Quintens R. (2014). Space radiation effects on plant and mammalian cells. *Acta Astronaut.* 104 419–431. 10.1016/j.actaastro.2014.05.005

[B10] Baby S. M. Narayanaswamy G. K. Anand A. (2011). Superoxide radical production and performance index of Photosystem II in leaves from magnetoprimed soybean seeds. *Plant Signal. Behav.* 6 1635–1637. 10.4161/psb.6.11.17720 22067104 PMC3329323

[B11] Balakhnina T. Bulak P. Nosalewicz M. Pietruszewski S. Włodarczyk T. (2015). The influence of wheat *Triticum aestivum* L. seed pre-sowing treatment with magnetic fields on germination, seedling growth, and antioxidant potential under optimal soil watering and flooding. *Acta Physiol. Plant.* 37:59 10.1007/s11738-015-1802-2

[B12] Balestrazzi A. Confalonieri M. Macovei A. Carbonera D. (2011). Seed imbibition in *Medicago truncatula* Gaertn: expression profiles of DNA repair genes in relation to PEG-mediated stress. *J. Plant Physiol.* 168 706–713. 10.1016/j.jplph.2010.10.008 21129815

[B13] Banik S. Bandyopadhyay S. Ganguly S. (2003). Bioeffects of microwave–a brief review. *Bioresour. Technol.* 87 155–159. 10.1016/S0960-8524(02)00169-4 12765354

[B14] Beard B. H. Haskins F. A. Gardner C. O. (1958). Comparison of effects of X-rays and thermal neutrons on dormant seeds of barley, maize, mustard, and saﬄower. *Genetics* 43 728–736. 17247791 10.1093/genetics/43.4.728 PMC1209915

[B15] Belyavskaya N. A. (2004). Biological effects due to weak magnetic field on plants. *Adv. Sp. Res.* 34 1566–1574. 10.1016/j.asr.2004.01.021 15880893

[B16] Belz R. G. Piepho H.-P. (2012). Modeling effective dosages in hormetic dose-response studies. *PLoS ONE* 7:e33432 10.1371/journal.pone.0033432 PMC3306408 22438929

[B17] Benedict H. M. Kersten H. (1934). Effect of soft X-rays on germination of wheat seeds. *Plant Physiol.* 9 173–178. 10.1104/pp.9.1.173 16652870 PMC439834

[B18] Bhardwaj J. Anand A. Nagarajan S. (2012). Biochemical and biophysical changes associated with magnetopriming in germinating cucumber seeds. *Plant Physiol. Biochem.* 57 67–73. 10.1016/j.plaphy.2012.05.008 22683465

[B19] Bilalis D. J. Katsenios N. Efthimiadou A. Karkanis A. Efthimiadis P. (2012). Investigation of pulsed electromagnetic field as a novel organic pre-sowing method on germination and initial growth stages of cotton. *Electromagn. Biol. Med.* 31 143–150. 10.3109/15368378.2011.624660 22268861

[B20] Bless A. A. (1938). Effects of X-rays on seeds. *Plant Physiol.* 13 209–211. 10.1104/pp.13.1.209 16653480 PMC438225

[B21] Borzouei A. Naseriyan B. Majdabadi A. Kafi M. Khazaei H. (2010). Effects of gamma radiation on germination and physiological aspects of wheat (*Triticum aestivum* L.) seedlings. *Pakistan J. Bot.* 42 2281–2290.

[B22] Brown J. E. Lu T. Y. Stevens C. Khan V. A. Lu J. Y. Wilson C. L. (2001). The effect of low dose ultraviolet light-C seed treatment on induced resistance in cabbage to black rot (*Xanthomonas campestris* pv. campestris). *Crop Prot.* 20 873–883. 10.1016/S0261-2194(01)00037-0

[B23] Buitink J. Claessens M. A. E. Hemminga M. A. Hoekstra F. A. (1998). Influence of water content and temperature on molecular mobility and intracellular glasses in seeds and pollen. *Plant Phys.* 118 531–554. 10.1104/pp.118.2.531 PMC34828 9765538

[B24] Buitink J. Hemminga M. A. Hoekstra F. A. (1999). Characterization of molecular mobility in seed tissues: an electron paramagnetic resonance spin probe study. *Biophys. J.* 76 3315–3322. 10.1016/S0006-3495(99)77484-9 10354457 PMC1300301

[B25] Buitink J. Leprince O. Hemminga M. A. Hoekstra F. A. (2000). Molecular mobility in the cytoplasm: an approach to describe and predict lifespan of dry germplasm. *Proc. Natl. Acad. Sci. U.S.A.* 97 2385–2390. 10.1073/pnas.040554797 10681458 PMC15810

[B26] Calabrese E. J. Baldwin L. A. (2000). Radiation hormesis: its historical foundations as a biological hypothesis. *Hum. Exp. Toxicol.* 19 41–75. 10.1191/096032700678815585 10745294

[B27] Caldecott R. S. Frolik E. F. Morris R. (1952). A comparison of the effects of X-rays and thermal neutrons on dormant seeds of barley. *Proc. Natl. Acad. Sci. U.S.A.* 38 804–809. 10.1073/pnas.38.9.804 16589180 PMC1063657

[B28] Celestino C. Picazo M. L. Toribio M. (2000). Inflluence of chronic exposure to an electromagnetic field on germination and early growth of *Quercus suber* seeds: preliminary study. *Electromagn. Biol. Med.* 19 115–120.

[B29] Choudhary K. K. Agrawal S. B. (2014). Ultraviolet-B induced changes in morphological, physiological and biochemical parameters of two cultivars of pea (*Pisum sativum* L.). *Ecotoxicol. Environ. Saf.* 100 178–187. 10.1016/j.ecoenv.2013.10.032 24268741

[B30] Confalonieri M. Faè M. Balestrazzi A. Donà M. Macovei A. Valassi A. (2014). Enhanced osmotic stress tolerance in *Medicago truncatula* plants overexpressing the DNA repair gene MtTdp2α (tyrosyl-DNA phosphodiesterase 2). *Plant Cell Tissue Organ. Cult.* 116 187–203. 10.1007/s11240-013-0395-y

[B31] De Micco V. Paradiso R. Aronne G. De Pascale S. Quarto M. Arena C. (2014). Leaf anatomy and photochemical behaviour of *Solanum lycopersicum* L. plants from seeds irradiated with low-LET ionising radiation. *Sci. World J.* 2014:428141 10.1155/2014/428141 PMC4030580 24883400

[B32] De Souza A. Garcí D. Sueiro L. Gilart F. Porras E. Licea L. (2006). Pre-sowing magnetic treatments of tomato seeds increase the growth and yield of plants. *Bioelectromagnetics* 27 247–257. 10.1002/bem.20206 16511881

[B33] Dubey A. K. Yadav J. R. Singh B. (2007). Studies on induced mutations by gamma irradiation in okra (*Abelmoschus esculentus* (L.) Monch.). *Progress. Agric.* 7 46–48.

[B34] Edmondson J. L. Davies Z. G. Gaston K. J. Leake J. R. (2014). Urban cultivation in allotments maintains soil qualities adversely affected by conventional agriculture. *J. Appl. Ecol.* 51 880–889. 10.1111/1365-2664.12254 25641978 PMC4301088

[B35] Efthimiadou A. Katsenios N. Karkanis A. Papastylianou P. Triantafyllidis V. Travlos I. (2014). Effects of presowing pulsed electromagnetic treatment of tomato seed on growth, yield, and lycopene content. *Sci. World J.* 2014:369745 10.1155/2014/369745 PMC4109073 25097875

[B36] Einset J. Collins A. R. (2015). DNA repair after X-irradiation: lessons from plants. *Mutagenesis* 30 45–50. 10.1093/mutage/geu054 25527727

[B37] Esnault M.-A. Legue F. Chenal C. (2010). Ionizing radiation: advances in plant response. *Environ. Exp. Bot.* 68 231–237. 10.1016/j.envexpbot.2010.01.007

[B38] Faè M. Balestrazzi A. Confalonieri M. Donà M. Macovei A. Valassi A. (2014). Copper-mediated genotoxic stress is attenuated by the overexpression of the DNA repair gene MtTdp2α (tyrosyl-DNA phosphodiesterase 2) in *Medicago truncatula* plants. *Plant Cell Rep.* 33 1071–1080. 10.1007/s00299-014-1595-6 24638978

[B39] Fan X. Toivonen P. N. Rajkowski K. T. Sokorai K. J. (2003). Warm water treatment in combination with modified atmosphere packaging reduces undesirable effects of irradiation on the quality of fresh-cut iceberg lettuce. *J. Agric. Food Chem.* 51 1231–1236. 10.1021/jf020600c 12590460

[B40] Farkas J. Mohácsi-Farkas C. (2011). History and future of food irradiation. *Trends Food Sci. Technol.* 22 121–126. 10.1016/j.tifs.2010.04.002

[B41] Farokh P. Mahmoodzadeh H. Satari T. (2010). Response of seed germination of saﬄower to UV-B radiation. *Res. J. Environ. Sci.* 4 70–74. 10.3923/rjes.2010.70.74

[B42] Ghodbane S. Lahbib A. Sakly M. Abdelmelek H. (2013). Bioeffects of static magnetic fields: oxidative stress, genotoxic effects, and cancer studies. *Biomed. Res. Int.* 2013:602987 10.1155/2013/602987 PMC3763575 24027759

[B43] Gicquel M. Taconnat L. Renou J. P. Esnault M. A. Cabello-Hurtado F. (2012). Kinetic transcriptomic approach revealed metabolic pathways and genotoxic-related changes implied in the *Arabidopsis* response to ionising radiations. *Plant Sci.* 195 106–119. 10.1016/j.plantsci.2012.06.015 22921004

[B44] Golovina E. A. Tikhonov A. N. Hoekstra F. A. (1997). An electron paramagnetic resonance spin-probe study of membrane permeability changes with seed ageing. *Plant Phys.* 114 383–389. 10.1104/pp.114.1.383 PMC158314 12223711

[B45] Guajardo-Flores D. Serna-Guerrero D. Serna-Saldívar S. O. Jacobo-Velázquez D. A. (2014). Effect of germination and UV-C radiation on the accumulation of flavonoids and saponins in black bean seed coats. *Cereal Chem.* 91 276–279. 10.1094/CCHEM-08-13-0172-R

[B46] Hamid N. Jawaid F. (2011). Influence of seed pre-treatment by UV-A and UV-C radiation on germination and growth of Mung beans. *Pakistan J. Chem.* 1 164–167. 10.15228/2011.v01.i04.p04

[B47] Hegazi A. Z. Hamideldin N. (2010). The effect of gamma irradiation on enhancement of growth and seed yield of okra [*Abelmoschus esculentus* (L.) Monech] and associated molecular changes. *J. Hortic. For.* 2 38–51.

[B48] Heisler G. M. Grant R. H. Gao W. Slusser J. R. (2003). Ultraviolet radiation and its impacts on agriculture and forests. *Agric. For. Meteorol.* 120 3–7. 10.1016/j.agrformet.2003.08.007

[B49] Hideg E. Jansen M. A. K. Strid A. (2013). UV-B exposure, ROS, and stress: inseparable companions or loosely linked associates? *Trends Plant Sci.* 18 107–115. 10.1016/j.tplants.2012.09.003 23084465

[B50] Hollósy F. (2002). Effects of ultraviolet radiation on plant cells. *Micron* 33 179–197. 10.1016/S0968-4328(01)00011-7 11567887

[B51] Hussain S. Zheng M. Khan F. Khaliq A. Fahad S. Peng S. (2015). Benefits of rice seed priming are offset permanently by prolonged storage and the storage conditions. *Sci. Rep.* 5:8101 10.1038/srep08101 PMC4309961 25631923

[B52] Irfaq M. Nawab K. (2001). Effect of gamma irradiation on some morphological characteristics of three wheat (*Triticum aestivum* L.) cultivars. *J. Biol. Sci.* 1 935–937. 10.3923/jbs.2001.935.937

[B53] Iuliana C. Caprita R. Giancarla V. Sorina R. (2013). Response of barley seedlings to microwaves at 2.45 GHz. *Sci. Pap. Anim. Sci. Biotechnol.* 46 185–191.

[B54] Jakubowski T. (2010). The impact of microwave radiations at different frequencies on the weight of seed potato germs and crop of potato tubers. *Agric. Eng.* 6 57–64.

[B55] Javed N. Ashraf M. Akram N. A. Al-Qurainy F. (2011). Alleviation of adverse effects of drought stress on growth and some potential physiological attributes in maize (*Zea mays* L.) by seed electromagnetic treatment. *Photochem. Photobiol.* 87 1354–1362. 10.1111/j.1751-1097.2011.00990.x 21883242

[B56] Jayasanka S. M. D. H. Asaeda T. (2014). The significance of microwaves in the environment and its effect on plants. *Environ. Rev.* 22 220–228. 10.1016/j.biortech.2015.02.055

[B57] Jayawardena S. D. L. Peiris R. (1988). Food crops breeding in Sri Lanka - achievements and challenges. *BIO News* 4 22–34.

[B58] Knox O. G. G. McHugh M. J. Fountaine J. M. Havis N. D. (2013). Effects of microwaves on fungal pathogens of wheat seed. *Crop Prot.* 50 12–16. 10.1016/j.cropro.2013.03.009

[B59] Kotwaliwale N. Singh K. Kalne A. Jha S. N. Seth N. Kar A. (2014). X-ray imaging methods for internal quality evaluation of agricultural produce. *J. Food Sci. Technol.* 51 1–15. 10.1007/s13197-011-0485-y 24426042 PMC3857400

[B60] Kovács E. Keresztes Á (2002). Effect of gamma and UV-B/C radiation on plant cells. *Micron* 33 199–210. 10.1016/S0968-4328(01)00012-9 11567888

[B61] Kovalchuk I. Molinier J. Yao Y. Arkhipov A. Kovalchuk O. (2007). Transcriptome analysis reveals fundamental differences in plant response to acute and chronic exposure to ionizing radiation. *Mutat. Res. Fund. Mol. M* 624 101–113. 10.1016/j.mrfmmm.2007.04.009 17568626

[B62] Krylov A. Tarakanova G. A. (1960). Magnetotropism of plants and its nature. *Plant Phys.* 7 156–160.

[B63] Kurdziel M. Dlubacz A. Weselucha-Birczynska A. Filek M. Labanowska M. (2015). Stable radicals and biochemical compounds in embryos and endosperm of wheat grains differentiating sensitive and tolerant genotypes-EPR and Raman studies. *J. Plant Phys.* 183 95–107. 10.1016/j.jplph.2015.05.018 26121078

[B64] Labanowska M. Filek M. Kurdziel M. Bednarska E. Dlubacz A. Hartikainen H. (2012). Electron paramagnetic resonance (EPR) spectroscopy characterization of wheat grains from plants of different water stress tolerance. *J. Plant Phys.* 169 1234–1242. 10.1016/j.jplph.2012.04.020 22840996

[B65] Labanowska M. Kurdziel M. Filek M. (2016). Changes of paramagnetic species in cereal grains upon short-term ozone action as a marker of oxidative stress tolerance. *J. Plant Physiol.* 190 54–66. 10.1016/j.jplph.2015.10.011 26655395

[B66] Leprince O. Deltour R. Thorpe P. C. Atherton N. M. Hendry G. A. F. (1990). The role of free radicals and radical processing systems in loss of desiccation tolerance in germinating maize (*Zea mays* L.). *New Phytol.* 116 573–580. 10.1111/j.1469-8137.1990.tb00541.x

[B67] Leprince O. Hoekstra F. A. (1998). The responses of cytochrome redox state and energy metabolism to dehydration support a role for cytoplasmic viscosity in desiccation tolerance. *Plant Physiol.* 118 1253–1264. 10.1104/pp.118.4.1253 9847099 PMC34741

[B68] Leprince O. Vertucci C. W. Hendry G. A. F. Atherton N. M. (1995). The expression of desiccation-induced damage in orthodox seeds is a function of oxygen and temperature. *Physiol. Plant.* 94 233–240. 10.1034/j.1399-3054.1995.940208.x

[B69] Luckey T. D. (1980). *Hormesis with Ionizing Radiation.* Boca Raton: CRC press.

[B70] Luckey T. D. (2006). Radiation hormesis: the good, the bad, and the ugly. *Dose Response.* 4 169–190. 10.2203/dose-response.06-102.Luckey 18648595 PMC2477686

[B71] Macovei A. Garg B. Raikwar S. Balestrazzi A. Carbonera D. Buttafava A. (2014). Synergistic exposure of rice seeds to different doses of γ -ray and salinity stress resulted in increased antioxidant enzyme activities and gene-specific modulation of TC-NER pathway. *Biomed Res. Int.* 170 780–787. 10.1155/2014/676934 PMC3914328 24551849

[B72] Maffei M. E. (2014). Magnetic field effects on plant growth, development, and evolution. *Front. Plant Sci.* 5:445 10.3389/fpls.2014.00445 PMC4154392 25237317

[B73] Maherchandani N. (1975). Effects of gamma radiation on the dormant seed of *Avena fatua* L. *Radiat. Bot.* 15 439–443. 10.1016/0033-7560(75)90018-6

[B74] Maity J. P. Mishra D. Chakraborty A. Saha A. Santra S. C. Chanda S. (2005). Modulation of some quantitative and qualitative characteristics in rice (*Oryza sativa* L.) and mung (*Phaseolus mungo* L.) by ionizing radiation. *Radiat. Phys. Chem.* 74 391–394. 10.1016/j.radphyschem.2004.08.005

[B75] Majeed A. Ahmad H. Muhammad Z. (2010). Variation in chlorophyll contents and grain yield of *Lepidium sativum* L as induced by gamma irradiation. *Int. J. Biol. Sci. Eng.* 1 147–151.

[B76] Marcu D. Damian G. Cosma C. Cristea V. (2013). Gamma radiation effects on seed germination, growth and pigment content, and ESR study of induced free radicals in maize (*Zea mays*). *J. Biol. Phys.* 39 625–634. 10.1007/s10867-013-9322-z 23996407 PMC3758825

[B77] Mattson M. P. (2008). Hormesis defined. *Ageing Res. Rev.* 7 1–7. 10.1016/j.arr.2007.08.007 18162444 PMC2248601

[B78] Minisi F. A. El-Mahrouk M. E. Rida M. E.-D. F. Nasr M. N. (2013). Effects of gamma radiation on germination, growth characteristics and morphological variations of *Moluccella laevis* L. *Am. Eurasian J. Agric. Environ. Sci.* 13 696–704.

[B79] Mitchell L. M. Cambrosio A. (1997). The invisible topography of power: electromagnetic fields, bodies and the environment. *Soc. Stud. Sci.* 27 221–271. 10.1177/030631297027002002

[B80] Mokobia C. E. Anomohanran O. (2005). The effect of gamma irradiation on the germination and growth of certain Nigerian agricultural crops. *J. Radiol. Prot.* 25 181–188. 10.1088/0952-4746/25/2/006 15942061

[B81] Moussa H. R. (2006). Role of gamma irradiation in regulation of NO3 level in rocket (*Eruca vesicaria* subsp. sativa) plants. *Russ. J. Plant Physiol.* 53 193–197. 10.1134/S1021443706020075

[B82] Ouhibi C. Attia H. Rebah F. Msilini N. Chebbi M. Aarrouf J. (2014). Salt stress mitigation by seed priming with UV-C in lettuce plants, growth, antioxidant activity and phenolic compounds. *Plant Phys. Biochem.* 83 126–133. 10.1016/j.plaphy.2014.07.019 25133899

[B83] Paparella S. Araújo S. S. Rossi G. Wijayasinghe M. Carbonera D. Balestrazzi A. (2015). Seed priming: state of the art and new perspectives. *Plant Cell Rep.* 34 1281–1293. 10.1007/s00299-015-1784-y 25812837

[B84] Pérez-Torres E. Kirchgessner N. Pfeifer J. Walter A. (2015). Assessing potato tuber diel growth by means of X-ray computed tomography. *Plant Cell Environ.* 38 2318–2326. 10.1111/pce.12548 25850677

[B85] Poinapen D. Brown D. C. W. Beeharry G. K. (2013). Seed orientation and magnetic field strength have more influence on tomato seed performance than relative humidity and duration of exposure to non-uniform static magnetic fields. *J. Plant Physiol.* 170 1251–1258. 10.1016/j.jplph.2013.04.016 23759543

[B86] Pretty J. N. Noble A. D. Bossio D. Dixon J. Hine R. E. Penning De Vries F. W. T. (2006). Resource-conserving agriculture increases yields in developing countries. *Environ. Sci. Technol.* 40 1114–1119. 10.1021/es051670d 16572763

[B87] Priestley D. A. Werner B. G. Leopold A. C. McBride M. B. (1985). Organic free radicals in seeds and pollen: the effect of hydration and ageing. *Physiol. Plant.* 64 88–94. 10.1111/j.1399-3054.1985.tb01217.x

[B88] Qi W. Zhang L. Wang L. Xu H. Jin Q. Jiao Z. (2015). Pretreatment with low-dose gamma irradiation enhances tolerance to the stress of cadmium and lead in *Arabidopsis thaliana* seedlings. *Ecotoxicol. Environ. Saf.* 115 243–249. 10.1016/j.ecoenv.2015.02.026 25723134

[B89] Ragonnaud M. (2013). *The EU seed and Plant Reproductive Material Market in Perspective: A Focus on Companies and Market Shares. Policy Department B: Structural and Cohesion Policies. European Parliament Committee on Agriculture and Rural Development*. Brussels: European Comission.

[B90] Rajendra P. Nayak H. S. Sashidhar R. B. Subramanyam C. Devendranath D. Gunasekaran B. (2005). Effects of power frequency electromagnetic fields on growth of germinating *Vicia faba* L., the broad bean. *Electromagn. Biol. Med.* 24 39–54. 10.1081/JBC-200055058

[B91] Reddy K. V. Reshma S. R. Jareena S. Nagaraju M. (2012). Exposure of greengram seeds (*Vigna radiate* var. radiata) to static magnetic fields: effects on germination and alfa-amylase activity. *Res. J. Seed Sci.* 5 106–114. 10.3923/rjss.2012.106.114

[B92] Reddy M. V. B. Kushalappa A. C. Raghavan G. S. V. Stephenson M. M. P. (1995). Use of microwave energy for the rradication of seedborne Diaporth phaseolorum in soybean and its effect on seed quality. *J. Microw. Power Electromagn. Energy* 30 199–204.

[B93] Reddy M. V. B. Raghavan G. S. V. Kushalappa A. C. Paulitz T. C. (1998). Effect of microwave treatment on quality of wheat seedsi with Fusarium graminearum. *J. Agric. Eng. Res.* 71 113–117. 10.1006/jaer.1998.0305

[B94] Sacandè M. Golovina E. A. Van Aelst A. C. Hoekstra F. A. (2001). Viability loss of neem (*Azadirachta indica*) seeds associated with membrane phase behaviour. *J. Exp. Bot.* 52 919–931. 10.1093/jexbot/52.358.919 11432909

[B95] Sahin H. (2014). Effects of microwaves on the germination of weed Seeds. *J. Biosyst. Eng.* 39 304–309. 10.5307/JBE.2014.39.4.304

[B96] Sahu I. D. McCarrik R. M. Lorigan G. A. (2013). Use of electron paramagnetic resonance to solve biochemical problems. *Biochemistry* 52 5967–5984. 10.1021/bi400834a 23961941 PMC3839053

[B97] Scialabba A. Tamburello C. (2002). Microwave effects on germination and growth of radish (*Raphanus sativus* L.) seedlings. *Acta Bot. Gall.* 149 113–123. 10.1080/12538078.2002.10515947

[B98] Shaukat S. Farooq M. Siddiqui M. Zaidi S. (2013). Effect of enhanced UV-B radiation on germination, seedling growth and biochemical responses of *Vigna mungo* (L.) Hepper. *Pak. J. Bot.* 45 779–785.

[B99] Shine M. B. Guruprasad K. N. Anand A. (2011). Enhancement of germination, growth, and photosynthesis in soybean by pre-treatment of seeds with magnetic field. *Bioelectromagnetics* 32 474–484. 10.1002/bem.20656 21381047

[B100] Siddiqui A. Dawar S. Javed Zaki M. Hamid N. (2011). Role of ultra violet (UV-C) radiation in the control of root infecting fungi on groundnut and mung bean. *Pakistan J. Bot.* 43 2221–2224.

[B101] Sjodin J. (1962). Some observations in X1 and X2 of *Vicia faba* L, after treatment with different mutagens. *Hereditas* 48 565–586.

[B102] Smirnov A. I. Golovina H. A. Yakimchenko O. E. Aksyonov S. J. Lebedev Y. S. (1992). In vivo seed investigation by electron paramagnetic resonance spin probe technique. *J. Plant Phys.* 140 447–452. 10.1016/S0176-1617(11)80823-0

[B103] Smith L. (1950). Effects of atomic bomb radiations and x-rays on seeds of cereals; a comparison of the effects of ionizing radiations from the “test Able” atomic bomb and from x-rays on seeds of barley, wheat and oats. *J. Hered.* 41 125–130. 15436968 10.1093/oxfordjournals.jhered.a106107

[B104] Soran M.-L. Stan M. Niinemets Ü. Copolovici L. (2014). Influence of microwave frequency electromagnetic radiation on terpene emission and content in aromatic plants. *J. Plant Physiol.* 171 1436–1443. 10.1016/j.jplph.2014.06.013 25050479 PMC4410321

[B105] Steffen-Heins A. Steffens B. (2015). EPR spectroscopy and its use in planta-a promising technique to disentangle the origin of specific ROS. *Front. Environ. Sci.* 3:15 10.3389/fenvs.2015.00015

[B106] Talei D. Valdiani A. Maziah M. Mohsenkhah M. (2013). Germination response of MR 219 rice variety to different exposure times and periods of 2450 MHz microwave frequency. *Sci. World J.* 2013:408026 10.1155/2013/408026 PMC3836452 24307869

[B107] Teixeira da Silva J. A. Dobránszki J. (2015). Magnetic fields: how is plant growth and development impacted? *Protoplasma* 253 231–248. 10.1007/s00709-015-0820-7 25952081

[B108] Uhlenbeck G. E. Goudsmit S. S. (1926). Spinning electrons and the structure of spectra. *Nature* 17 264–265. 10.1038/117264a0

[B109] Vashisth A. Nagarajan S. (2010). Effect on germination and early growth characteristics in sunflower (*Helianthus annuus*) seeds exposed to static magnetic field. *J. Plant Physiol.* 167 149–156. 10.1016/j.jplph.2009.08.011 19783321

[B110] Velázquez-Martí B. Gracia-López C. Marzal-Domenech A. (2006). Germination inhibition of undesirable seed in the soil using microwave radiation. *Biosyst. Eng.* 93 365–373. 10.1016/j.biosystemseng.2006.01.005

[B111] Ventura L. Donà M. Macovei A. Carbonera D. Buttafava A. Mondoni A. (2012). Understanding the molecular pathways associated with seed vigor. *Plant Physiol. Biochem.* 60 196–206. 10.1016/j.plaphy.2012.07.031 22995217

[B112] Vian A. Roux D. Girard S. Bonnet P. Paladian F. Davies E. (2006). Microwave irradiation affects gene expression in plants. *Plant Signal. Behav.* 1 67–70. 10.4161/psb.1.2.2434 19521478 PMC2633881

[B113] Weil J. A. Bolton J. R. (2006). *Electron Paramagnetic Resonance: Elementary, Theory and Practical Applications*. Hoboken, NJ: John Wiley and Sons, Inc.

[B114] Wolff S. A. Coelho L. H. Karoliussen I. Jost A.-I. K. (2014). Effects of the extraterrestrial Environment on plants: recommendations for future space experiments for the MELiSSA higher plant compartment. *Life* 4 189–204. 10.3390/life4020189 25370192 PMC4187168

[B115] Yagyu P. Morris R. (1957). Cytogenetic effects of X-rays and thermal neutrons on dormant tomato seeds. *Genetics* 42 222–238. 17247692 10.1093/genetics/42.3.222 PMC1209826

[B116] Zaka R. Chenal C. Misset M. (2002). Study of external low irradiation dose effects on induction of chromosome aberrations in *Pisum sativum* root tip meristem. *Mutat. Res. Toxicol. Environ. Mutagen.* 517 87–99. 10.1016/S1383-5718(02)00056-6 12034311

[B117] Zavoisky E. (1945). Spin-magnetic resonance in paramagnetics. *J. Physics-USSR* 9 245.

[B118] Zeeman P. (1897). The effect of magnetisation on the nature of light emitted by a substance. *Nature* 55:347 10.1038/055347a0

